# Truncated recombinant human SP-D attenuates emphysema and type II cell changes in SP-D deficient mice

**DOI:** 10.1186/1465-9921-8-70

**Published:** 2007-10-03

**Authors:** Lars Knudsen, Matthias Ochs, Rosemarie MacKay, Paul Townsend, Roona Deb, Christian Mühlfeld, Joachim Richter, Fabian Gilbert, Samuel Hawgood, Kenneth Reid, Howard Clark

**Affiliations:** 1 Department of Anatomy, Division of Electron Microscopy, University of Göttingen, Göttingen, Germany; 2 Institute of Anatomy, Experimental Morphology, University of Bern, Bern, Switzerland; 3 MRC Immunochemistry Unit, Department of Biochemistry, University of Oxford, Oxford, UK; 4 Institute of Anatomy, Department of Histology, University of Bern, Bern, Switzerland; 5 Department of Pediatrics and Cardiovascular Research Institute, University of California, San Francisco, CA, USA; 6 Department of Child Health, University of Southampton, Southampton General Hospital, Southampton, UK

## Abstract

**Background:**

Surfactant protein D (SP-D) deficient mice develop emphysema-like pathology associated with focal accumulations of foamy alveolar macrophages, an excess of surfactant phospholipids in the alveolar space and both hypertrophy and hyperplasia of alveolar type II cells. These findings are associated with a chronic inflammatory state. Treatment of SP-D deficient mice with a truncated recombinant fragment of human SP-D (rfhSP-D) has been shown to decrease the lipidosis and alveolar macrophage accumulation as well as production of proinflammatory chemokines. The aim of this study was to investigate if rfhSP-D treatment reduces the structural abnormalities in parenchymal architecture and type II cells characteristic of SP-D deficiency.

**Methods:**

SP-D knock-out mice, aged 3 weeks, 6 weeks and 9 weeks were treated with rfhSP-D for 9, 6 and 3 weeks, respectively. All mice were sacrificed at age 12 weeks and compared to both PBS treated SP-D deficient and wild-type groups. Lung structure was quantified by design-based stereology at the light and electron microscopic level. Emphasis was put on quantification of emphysema, type II cell changes and intracellular surfactant. Data were analysed with two sided non-parametric Mann-Whitney U-test.

**Main Results:**

After 3 weeks of treatment, alveolar number was higher and mean alveolar size was smaller compared to saline-treated SP-D knock-out controls. There was no significant difference concerning these indices of pulmonary emphysema within rfhSP-D treated groups. Type II cell number and size were smaller as a consequence of treatment. The total volume of lamellar bodies per type II cell and per lung was smaller after 6 weeks of treatment.

**Conclusion:**

Treatment of SP-D deficient mice with rfhSP-D leads to a reduction in the degree of emphysema and a correction of type II cell hyperplasia and hypertrophy. This supports the concept that rfhSP-D might become a therapeutic option in diseases that are characterized by decreased SP-D levels in the lung.

## Background

Pulmonary emphysema or COPD is a common disease for which there is currently no effective therapy. The WHO estimates that COPD is the fifth leading cause of death worldwide (WHO world health report 2002) and the prevalence and mortality are expected to increase in the coming decades [1]. Several studies demonstrated, that surfactant protein D (SP-D) levels are diminished in the lung of smokers or cystic fibrosis patients [2-5]. Another study identified COPD susceptibility-alleles in the gene-location of the surfactant proteins, suggesting their role in the pathogenesis of COPD [6]. SP-D, along with surfactant protein A (SP-A) belong to the collectin family of mammalian C-type lectins and are known to be important innate host defense molecules at mucosal surfaces, with a recognized role in controlling inflammation [7]. As reviewed by Hartl and Griese recently, the anti-inflammatory properties of SP-D seem to be of importance in many human lung diseases [8]. Ablation of the gene for SP-D in mice provided evidence that SP-D protects the normal murine lung from chronic pulmonary inflammation and emphysema since even in the absence of any infectious challenge, SP-D-deficiency causes the spontaneous development of emphysema-like pathology. The lungs of mice deficient in SP-D exhibit hypertrophy and hyperplasia of type II cells, a diminished number of alveoli, increased alveolar size and decreased alveolar surface area [9]. Surfactant homeostasis is disturbed, indicated by the presence of giant lamellar bodies in some type II cells and the development of alveolar lipoproteinosis [10]. In addition to the increased intra-alveolar surfactant pool, stereological analysis revealed an increased intracellular surfactant pool [9].

We have previously reported that a considerable number of apoptotic and necrotic alveolar macrophages are present in the bronchoalveolar lavage (BAL) in SP-D knock-out mice [11] and have postulated that delayed clearance of dead and dying cells may be involved in generating a chronic inflammatory state which leads to emphysema [12-15]. The chronic alveolar macrophage mediated inflammatory state is characterized by increased numbers of alveolar macrophages in the alveolar space, high levels of reactive oxygen species (ROS) and raised expression of matrix metalloproteinases (MMP) [16,17]. Treatment with a recombinant fragment of human SP-D (rfhSP-D) was sufficient to reduce the numbers of dead and dying alveolar macrophages, production of proinflammatory chemokines as well as alveolar lipoproteinosis [14]. Thus agents such as rfhSP-D may be protective against lung remodeling and destruction due to their ability to reduce numbers of apoptotic and necrotic cells.

We therefore hypothesized that treatment with rfhSP-D would lead to an attenuation of the structural alterations present in SP-D-deficient mice. To test this, we quantified the degree of pulmonary emphysema, type II cell alterations and the intracellular surfactant pool by design-based stereology at the light and electron microscopic level [18] in lungs of mice, which were treated with rfhSP-D. Our results imply that intranasal application of rfhSP-D is effective in preventing structural abnormalities characteristic of SP-D-deficiency.

## Methods

### Preparation of rfhSP-D

The recombinant fragment of SP-D was expressed in E coli and purified as described in detail elsewhere [19]. Briefly, the cDNA for the neck/CRD, including a short region of the collagen stalk (8 Gly-X-Y) and representing residues 179–355 was cloned from human lung library DNA and inserted into a pET-21d vector (Novagen, Nottingham). The plasmid was transformed into BL21(λDE3) pLysS and a single colony selected and re-plated to give 100–400 colonies/plate. These were scraped and used to inoculate shake-flasks containing 500 ml LB medium supplemented with 100 μg/ml ampicillin and 25 μg/ml chloramphenicol and grown to an OD600 of 0.6–0.8 followed by induction with 0.4 mM IPTG for 2–3 hours. Cells were collected by centrifugation and lysed in 20 mM Tris-HCl, 150 mM NaCl, 5 mM EDTA, 0.1% v/v Triton X-100, 0.1 mM PMSF, pH 7.5 and sonicated for 3 minutes. The rfhSP-D is expressed in insoluble inclusion bodies and was collected by centrifugation and washed 4 times at 10000 × g. The pellet was solubilized in 100 ml of 8 M Urea, 100 mM 2-mercaptoethanol, pH 7.5 and clarified by centrifugation and refolded by overnight dialysis against 10 L of 20 mM Tris-HCl, 150 mM NaCl, 5 mM CaCl_2 _(TCB). Refolded rfhSP-D was separated from denatured rfhSP-D by absorption onto maltose-agarose (Sigma-Aldrich, Poole, UK) and eluted with 20 mM Tris-HCl, 150 mM NaCl, containing 5 mM EDTA after first washing the column with TCB containing 1 M NaCl to remove impurities. Final purification was by gel filtration column (Superose 12, Amersham Pharmacia, UK) in a running buffer of 20 mM Tris-HCl, 150 mM NaCl, 5 mM EDTA, 0.02% (w/v) sodium azide pH7.4 (TSE). The rfhSP-D eluted as a single peak corresponding to 60 kDa molecular weight. The recombinant preparation was judged to be pure by using SDS-PAGE, immunoblotting, and amino-terminal sequencing. The purified trimeric recombinant protein was assessed for correct folding by disulfide mapping and by its crystallographic structure complexed with maltose in the carbohydrate-binding pockets [20]. Endotoxin levels were reduced by passing the purified rfhSP-D through a 10 ml Polymixin B column (Detoxi-Gel, Pierce & Warriner, UK) and only preparations containing less than 5 pg/μg were used.

### Study subjects and administration of recombinant fragment SP-D

SP-D knock-out mice [10], back-crossed 10 generations into a C57BL/6 background were fed ad libitum and housed in isolators in a pathogen free environment in the Biomedical Services Unit at Oxford University. Pathogen free C57BL/6 wild-type controls were obtained from Harlan-OLAC (Shaw's Farm, Bicester, Oxfordshire, UK). Five groups of mice were investigated. Each group consisted of four to five animals. All mice included in this study were 12 weeks old when sacrificed. The animals of groups D3, D6 and D9 were subjected to an intranasal treatment with rfhSP-D. Five doses of 10 μg rfhSP-D in 50 μl PBS were administrated per week, one dose per day from Monday to Friday. As it has been shown previously, 40% of the administrated rfhSP-D could be found in cell free BAL one hour after application [11]. This corresponds to the concentration of native SP-D in WT group. After 21 hours no rfhSP-D could be detected in the BAL [11].

The group D0 only received PBS from week 3 to week 12 and no rfhSP-D. The group D3 received therapy from the age of 9 weeks, D6 from the age of 6 weeks and D9 from the age of 3 weeks (Table 1). Animals of the group which only contained wild-type mice (WT) neither got rfhSP-D nor PBS. All experimental protocols were approved by appropriate U.K. Home-Office licensing authorities and by the University of Oxford Ethical Committee.

**Table 1:** Definition of the groups

	**D0**	**D3**	**D6**	**D9**	**WT**
**genotype**	SP-D -/-	SP-D -/-	SP-D -/-	SP-D -/-	wild-type
**treatment**	PBS	rfhSP-D	rfhSP-D	rfhSP-D	-
**Number of animals**	5	4	5	5	4
**age at first application (weeks)**	3	9	6	3	-
**duration of treatment (weeks)**	9	3	6	9	-

### Fixation, sampling and processing

At age 12 weeks in all cases, the lungs were instillation fixed at a hydrostatic pressure of 20 cm H_2_O, using a 1.5% glutaraldehyde/1.5% paraformaldehyde mixture in 0.15 M Hepes buffer. After storage of the lungs in fresh fixative for at least 2 hours, the lungs were sampled for stereological analysis [18]. The total lung volume (V(lung)) was determined by means of the fluid displacement method [21]. In order to give every part of the lung an equal chance of being included in the stereological analysis and thereby represent the whole organ equally well, a systematic uniform random sampling design was applied and at least four samples per lung were taken and processed for light and electron microscopy [22].

By means of a tissue slicer, each organ was cut into 10 to 12 horizontal slices of 3 mm thickness. Starting with a random number, every other slice was chosen for light or electron microscopy, respectively. For light microscopy, the entire slices were subsequently osmicated, immersed in half-saturated watery uranyl acetate, dehydrated in acetone and embedded in glycol methacrylate (Technovit 7100, Heraeus Kulzer, Wehrheim, Germany). For electron microscopy, a transparent point grid was projected onto the sampled slices. Whenever a grid point hit the cut surface of a lung slice, tissue blocks were excised. By this method 8–10 blocks were obtained from each single lung. The tissue blocks were postfixed in osmium tetroxide, stained en bloc in half-saturated watery uranyl acetate, dehydrated in an ascending acetone series and embedded in araldite (SERVA Electrophoresis GmbH, Heidelberg, Germany). Three of the araldite blocks were randomly sampled for ultrastructural analysis.

### Stereological analysis

At the light microscopic level the stereological analysis was carried out using an Axioskop light microscope (Zeiss, Oberkochen, Germany) combined with a computer-assisted stereology system (CAST 2.0; Olympus, Ballerup, Denmark). From each lung, sections of 3 to 4 tissue blocks were analyzed. From each block embedded in glycol methacrylate, sections were cut with a thickness of 1.5 μm. The first and the third section of a consecutive row of sections were mounted on one glass slide, so that a distance from the top of the first to the top of third section of 3 μm resulted. Afterwards an orcein staining was performed. By means of point counting on the first of the parallel sections the volume fraction of parenchyma within the lung (V_v_(par/lung)) was determined. The estimation of the alveolar number (N(alv,lung)) was conducted by means of a physical disector principle using the two sections for counting both ways, i.e. using each section once as sampling section for counting and once as look-up section for comparison. That allowed us to determine the Euler number of the network of alveolar openings [18,23,24]. The orcein staining of the elastic fibres of the alveolar openings was required to distinguish between real alveolar openings and artefacts or pores of Kohn respectively. Division of the total number of alveoli per lung by the volume of parenchyma brings about the numerical density of alveoli (N_V_(alv/par)).

From each lung semi-thin sections of four blocks embedded in araldite were cut with a thickness of 1 μm. The first and the fourth section of a consecutive row of cuts were mounted on one glass slide parallely and stained with methylene blue. By means of point counting and intersection counting on the first of the parallel sections the volume fraction of both distal airspace (V_v_(air/par)) and septal tissue (V_v_(sep/par)) within parenchyma as well as the surface area of alveolar epithelium (S(alvepi,lung)) and the mean thickness of alveolar septa (τ¯
 MathType@MTEF@5@5@+=feaafiart1ev1aaatCvAUfKttLearuWrP9MDH5MBPbIqV92AaeXatLxBI9gBaebbnrfifHhDYfgasaacH8akY=wiFfYdH8Gipec8Eeeu0xXdbba9frFj0=OqFfea0dXdd9vqai=hGuQ8kuc9pgc9s8qqaq=dirpe0xb9q8qiLsFr0=vr0=vr0dc8meaabaqaciaacaGaaeqabaqabeGadaaakeaaiiGacuWFepaDgaqeaaaa@2E90@ (sep)) was determined according to established methods [18,25]. The mean alveolar volume (υ¯
 MathType@MTEF@5@5@+=feaafiart1ev1aaatCvAUfKttLearuWrP9MDH5MBPbIqV92AaeXatLxBI9gBaebbnrfifHhDYfgasaacH8akY=wiFfYdH8Gipec8Eeeu0xXdbba9frFj0=OqFfea0dXdd9vqai=hGuQ8kuc9pgc9s8qqaq=dirpe0xb9q8qiLsFr0=vr0=vr0dc8meaabaqaciaacaGaaeqabaqabeGadaaakeaaiiGacuWFfpqDgaqeaaaa@2E92@_N_(alv)) was determined by dividing the total volume of distal airspace by the total number of alveoli per lung.

Applying the physical disector principle [26] the number of type II cells per lung (N(typeII,lung)) was estimated. The disector height was 3 μm. In order to estimate the mean volume of type II cells (υ¯
 MathType@MTEF@5@5@+=feaafiart1ev1aaatCvAUfKttLearuWrP9MDH5MBPbIqV92AaeXatLxBI9gBaebbnrfifHhDYfgasaacH8akY=wiFfYdH8Gipec8Eeeu0xXdbba9frFj0=OqFfea0dXdd9vqai=hGuQ8kuc9pgc9s8qqaq=dirpe0xb9q8qiLsFr0=vr0=vr0dc8meaabaqaciaacaGaaeqabaqabeGadaaakeaaiiGacuWFfpqDgaqeaaaa@2E92@_N_(typeII)), the planar rotator was used [27]. The division of the total number of type II cells by the volume of parenchyma leads to the numerical density of alveolar type II cells (N_V_(typeII/par)).

At the electron microscopic level ultrathin sections of three blocks per lung were generated. At least 100 type II cells per lung were chosen by systematic uniform random sampling and analyzed. By point counting the volume fraction of lamellar bodies within type II cells (V_v_(lb/typeII)) was determined. The intracellular surfactant pool per cell, defined by morphological criteria as the total volume of lamellar bodies per type II cell (V(lb,typeII)), was calculated by multiplication of V_v_(lb/typeII) and υ¯
 MathType@MTEF@5@5@+=feaafiart1ev1aaatCvAUfKttLearuWrP9MDH5MBPbIqV92AaeXatLxBI9gBaebbnrfifHhDYfgasaacH8akY=wiFfYdH8Gipec8Eeeu0xXdbba9frFj0=OqFfea0dXdd9vqai=hGuQ8kuc9pgc9s8qqaq=dirpe0xb9q8qiLsFr0=vr0=vr0dc8meaabaqaciaacaGaaeqabaqabeGadaaakeaaiiGacuWFfpqDgaqeaaaa@2E92@_N_(typeII). Accordingly, the intracellular surfactant content per lung (V(lb,lung)) was determined by multiplication of V(lb,typeII) and N(typeII,lung). Furthermore, the volume-weighted mean volume of lamellar bodies (υ¯
 MathType@MTEF@5@5@+=feaafiart1ev1aaatCvAUfKttLearuWrP9MDH5MBPbIqV92AaeXatLxBI9gBaebbnrfifHhDYfgasaacH8akY=wiFfYdH8Gipec8Eeeu0xXdbba9frFj0=OqFfea0dXdd9vqai=hGuQ8kuc9pgc9s8qqaq=dirpe0xb9q8qiLsFr0=vr0=vr0dc8meaabaqaciaacaGaaeqabaqabeGadaaakeaaiiGacuWFfpqDgaqeaaaa@2E92@_v_(lb)) was estimated using the point sampled intercepts method [28].

For each parameter estimated, an observed coefficient of variance (CV_obs_) within a group was determined. This variance is composed of the biological variance due to given inter-individual differences within a population (CV_biol_) and an error provided by the stereological method (CE). Therefore the so called coefficient of error (CE) for each stereological parameter and animal was calculated (data not shown) [29,30]. The precision of stereological methods in this study was considered sufficient if the CE was not the major factor contributing to the CV_obs_, meaning that the given biological variance of the population is mostly responsible for the observed variance [25].

### Statistics

Data were analysed with the two sided non-parametric Mann-Whitney U-test. Tests were performed using the Statistica 6.0 software (StatSoft, Hamburg, Germany). A value of p < 0.05 was considered significant.

## Results

### Light and electron microscopy

**Figure 1:**
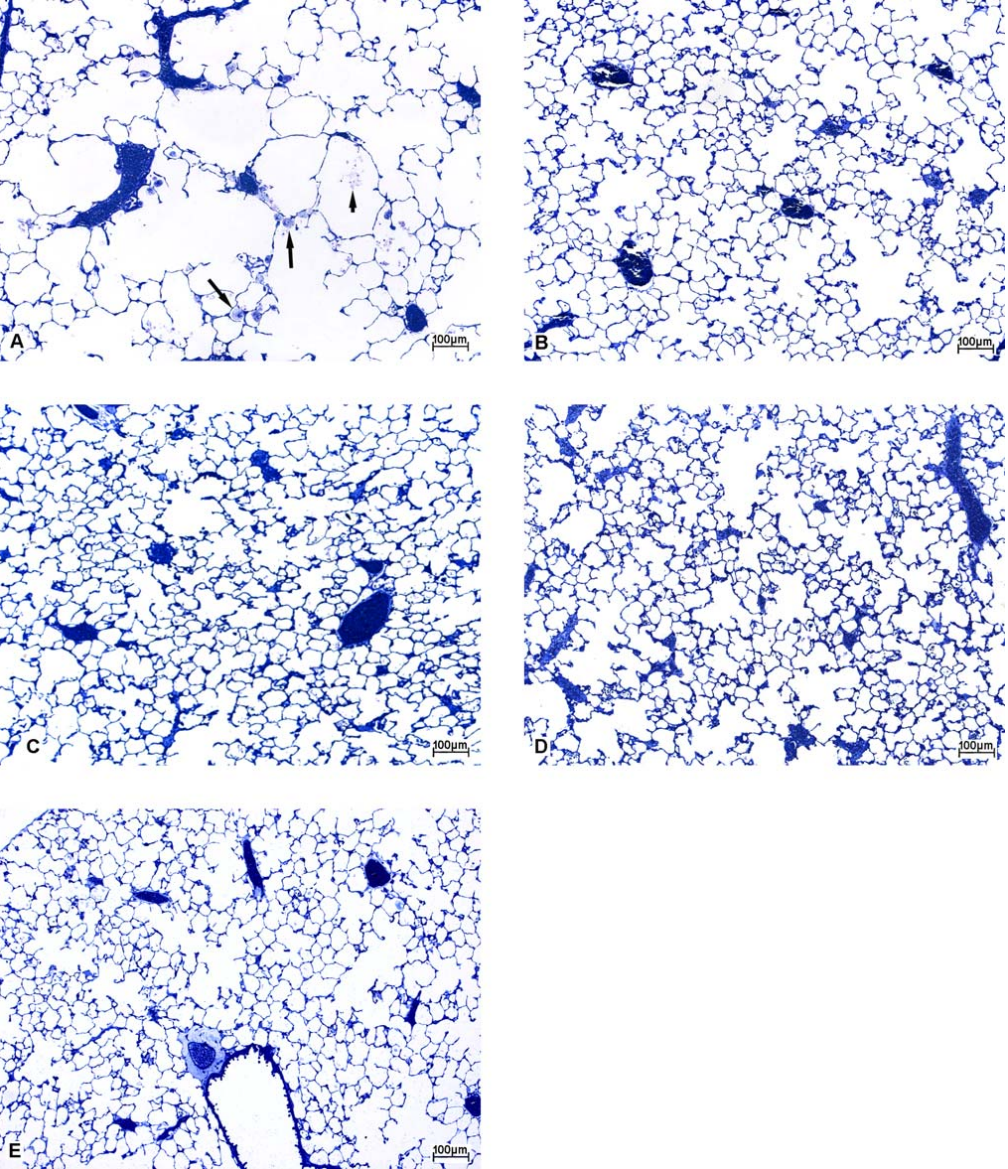
Low power light micrographs (methylene blue stained sections) from lungs of (A) group D0, (B) group D3, (C) group D6, (D) group D9 and (E) group WT. In contrast to untreated knock-out mice (A) the lungs of treated groups (B-D) demonstrate, compared to group WT (E), almost normal lung architecture with no major differences among them. In A an accumulation of intraalveolar surfactant (short arrow) and some foamy alveolar macrophages (long arrows) are visible.

The parenchymal architecture of the knock-out mice that were not treated with rfhSP-D but with PBS demonstrated typical features of SP-D-deficiency: the distal airspace was enlarged and there were focal accumulations of foamy alveolar macrophages particularly in the peri-bronchial and sub-pleural regions (Fig. 1A). In general, the alterations of the parenchymal architecture appeared much improved in large areas of the lung as a consequence of the treatment with rfhSP-D (Fig. 1B–D) and resembled the architecture of wild-type mice (Fig. 1E). This was clear after treatment for only three weeks (Fig. 1B). Nevertheless, in small areas, especially in the sub-pleural regions, emphysematous alterations were still evident, independent of the duration of treatment. Moreover, small accumulations of foamy alveolar macrophages were present in all treated groups.

**Figure 2:**
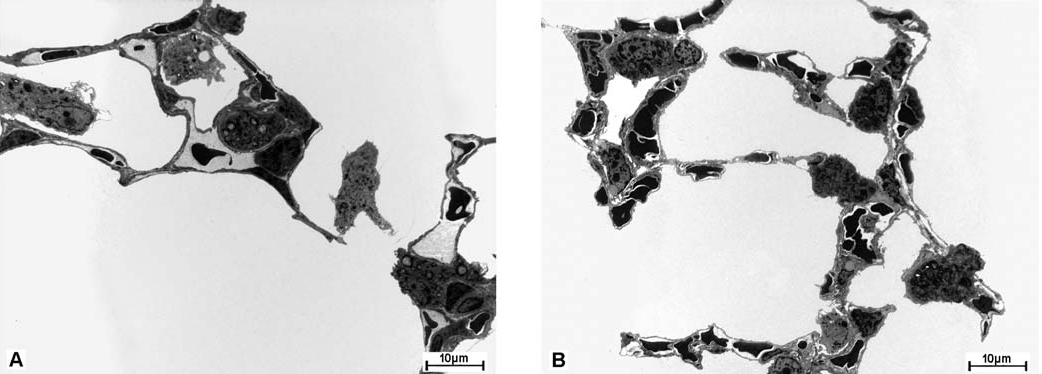
Low power electron micrographs from lungs of (A) group D0 and (B) group D3. Figure A demonstrates enlarged type II cells filled with abundant lamellar bodies and some alveolar macrophages. Compared to the untreated lung, B shows numerous type II cells without any obvious differences in size and lamellar body content per cell, emphasizing the need of proper design-based stereological investigation.

With respect to type II cell alterations, typical findings were present in the lungs of mice which were not treated with rfhSP-D. The type II cells were more numerous and enlarged (Fig. 2A). Giant lamellar bodies were occasionally observed. After a period of 3 weeks of treatment no obvious effect on the number of type II cells could be found. In several areas of the lungs the number of type II cells even seemed to have increased, the size of type II cells appeared to be unchanged (Fig. 2B). However, after 9 weeks of therapy, there was a clear decrease in the number of type II cells, though the size remained unaffected. Giant lamellar bodies were a rarity in all rfhSP-D treated groups. All these parameters were formally quantified stereologically as shown below.

### Stereological analyses

**Figure 3:**
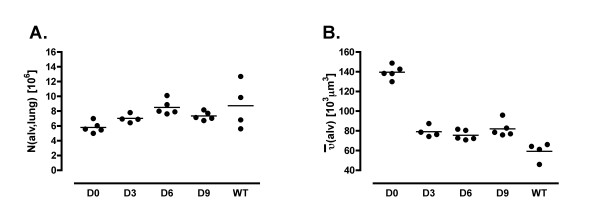
A: Number of alveoli per lung. B: Mean alveolar volume. Already after 3 weeks of treatment an increase in alveolar number and a decrease in alveolar volume was observed.

**Figure 4:**
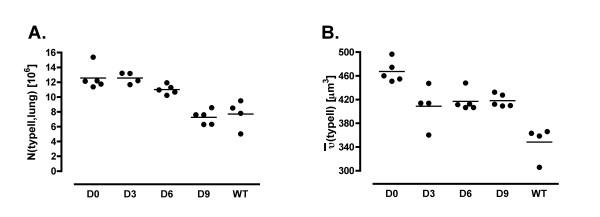
A: Number of type II cells per lung. B: Mean volume of type II cells. Whereas only after 6 weeks of treatment a slight decrease in type II cell number was found the cellular volume was already reduced after 3 weeks. The reduction of cell number went on after 9 weeks of treatment.

**Figure 5:**
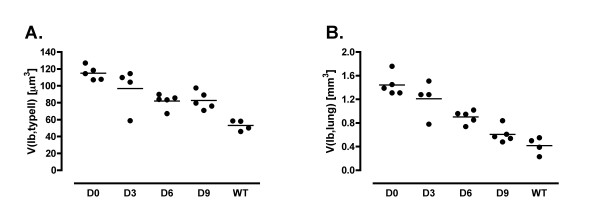
A: Volume of lamellar bodies per cell. B: Volume of lamellar bodies per lung. The content of lamellar bodies per cell and lung subsided significantly only after 6 weeks of treatment. A longer period of treatment led to a further decrease in lamellar body volume per lung but not per cell.

The stereological results are summarized in Table 2 and illustrated in Figures 3, 4, 5. With respect to the total lung volume, all rfhSP-D treated groups (D3, D6 and D9) demonstrated a significant reduction of 23% on average compared to D0 but did not differ from the WT group.

**Table 2:** Summarized stereological data

Parameter	**D0**	**D3**	**D6**	**D9**	**WT**
**V(lung) [cm^3^]**	1.00 (0.06)	0.72 (0.02)^†^	0.82 (0.07)^†^	0.76 (0.1)^†^	0.68 (0.22)^†^
**N(alv,lung) [10^6^]**	5.80 (0.52)	7.03 (0.58)^†^	8.51 (1.01)^†^	7.36 (0.58)^†^	8.75 (3.18)
**N_V_(alv/par) [10^3^/mm^3^]**	6.65 (0.58)	10.71 (0.51)^†^	11.17 (0.92)^†^	10.81 (0.95)^†^	14.11 (0.76)^†||§**^
υ¯ MathType@MTEF@5@5@+=feaafiart1ev1aaatCvAUfKttLearuWrP9MDH5MBPbIqV92AaeXatLxBI9gBaebbnrfifHhDYfgasaacH8akY=wiFfYdH8Gipec8Eeeu0xXdbba9frFj0=OqFfea0dXdd9vqai=hGuQ8kuc9pgc9s8qqaq=dirpe0xb9q8qiLsFr0=vr0=vr0dc8meaabaqaciaacaGaaeqabaqabeGadaaakeaaiiGacuWFfpqDgaqeaaaa@2E92@_N_**(alv) [10^3 ^μm^3^]**	139.67 (6.87)	79.26 (5.78)^†^	75.66 (5.1)^†^	82.07 (8.19)^†^	59.40 (9.19)^†||§**^
**S(alvepi,lung) [cm^2^]**	597.7 (51.9)	583.3 (66.4)	671.5 (74.1)	592.8 (61.2)	575.3 (157.2)
**S_V_(alvepi/par) [1/cm]**	661.8 (46.3)	910.3 (65.7)^†^	881.1 (47.1)^†^	866.6 (38.0)^†^	959.5 (146.3)^†^
**V_V_(air/par) [%]**	90.4 (2.3)	86.8 (3.1)	84.2 (3.0)^†^	88.2 (4.1)	83.5 (11.1)
**V_V_(sep/par) [%]**	10.4 (0.5)	13.8 (1.7)^†^	13.6 (1.5)^†^	13.4 (1.8)^†^	17.3 (7.2)^†^
τ¯ MathType@MTEF@5@5@+=feaafiart1ev1aaatCvAUfKttLearuWrP9MDH5MBPbIqV92AaeXatLxBI9gBaebbnrfifHhDYfgasaacH8akY=wiFfYdH8Gipec8Eeeu0xXdbba9frFj0=OqFfea0dXdd9vqai=hGuQ8kuc9pgc9s8qqaq=dirpe0xb9q8qiLsFr0=vr0=vr0dc8meaabaqaciaacaGaaeqabaqabeGadaaakeaaiiGacuWFepaDgaqeaaaa@2E90@ (sep) [μm]	3.14 (0.22)	3.04 (0.58)	3.08 (0.46)	3.08 (0.4)	3.5 (0.86)
**N(typeII,lung) [10^6^]**	12.57 (1.61)	12.58 (0.76)	11.02 (0.64)^†^	7.29 (0.97)^†||§^	7.73 (1.91)^†||§^
**N_V_(typeII/par) [10^3^/mm^3^]**	13.87 (1.12)	19.74 (1.58)^†^	14.52 (0.62)^||^	10.6 (0.59)^†||§^	13.15 (3.54)^||^
υ¯ MathType@MTEF@5@5@+=feaafiart1ev1aaatCvAUfKttLearuWrP9MDH5MBPbIqV92AaeXatLxBI9gBaebbnrfifHhDYfgasaacH8akY=wiFfYdH8Gipec8Eeeu0xXdbba9frFj0=OqFfea0dXdd9vqai=hGuQ8kuc9pgc9s8qqaq=dirpe0xb9q8qiLsFr0=vr0=vr0dc8meaabaqaciaacaGaaeqabaqabeGadaaakeaaiiGacuWFfpqDgaqeaaaa@2E92@_N_**(typeII) [μm^3^]**	467.5 (18.6)	408.97 (36.1)^†^	417.15 (17.5)^†^	418.38 (10.9)^†^	348.5 (28.53)^†||§**^
**V_V_(lb/typeII) [%]**	24.7 (2.5)	23.5 (4.4)	19.7 (1.9)^†^	19.8 (2.1)^†^	15.3 (1)^†||§**^
**V(lb,typeII) [μm^3^]**	114.9 (8.3)	97.0 (25.7)	82.1 (8.7)^†^	82.7 (10.7)^†^	53.2 (6.2)^†||§**^
**V(lb,lung) [mm^3^]**	1.44 (0.19)	1.21 (0.31)	0.9 (0.11)^†^	0.61 (0.14)^†||§^	0.42 (0.14)^†||§^
υ¯ MathType@MTEF@5@5@+=feaafiart1ev1aaatCvAUfKttLearuWrP9MDH5MBPbIqV92AaeXatLxBI9gBaebbnrfifHhDYfgasaacH8akY=wiFfYdH8Gipec8Eeeu0xXdbba9frFj0=OqFfea0dXdd9vqai=hGuQ8kuc9pgc9s8qqaq=dirpe0xb9q8qiLsFr0=vr0=vr0dc8meaabaqaciaacaGaaeqabaqabeGadaaakeaaiiGacuWFfpqDgaqeaaaa@2E92@_V_**(lb) [μm^3^]**	0.93 (0.12)	1.40 (0.35)	0.67 (0.08)^†||^	0.78 (0.03)^||^	0.48 (0.08)^†||§**^

Summarized stereological data, grouped into parameters related to parenchymal architecture, to type II cells, and to lamellar bodies. Values are given as mean (SD) of n = 4–5 mice per group. Abbreviations: V = volume, V_V _= volume density, S = surface area, S_V _= surface area density, τ¯
 MathType@MTEF@5@5@+=feaafiart1ev1aaatCvAUfKttLearuWrP9MDH5MBPbIqV92AaeXatLxBI9gBaebbnrfifHhDYfgasaacH8akY=wiFfYdH8Gipec8Eeeu0xXdbba9frFj0=OqFfea0dXdd9vqai=hGuQ8kuc9pgc9s8qqaq=dirpe0xb9q8qiLsFr0=vr0=vr0dc8meaabaqaciaacaGaaeqabaqabeGadaaakeaaiiGacuWFepaDgaqeaaaa@2E90@ = mean thickness, N = number, N_V _= numerical density, υ¯
 MathType@MTEF@5@5@+=feaafiart1ev1aaatCvAUfKttLearuWrP9MDH5MBPbIqV92AaeXatLxBI9gBaebbnrfifHhDYfgasaacH8akY=wiFfYdH8Gipec8Eeeu0xXdbba9frFj0=OqFfea0dXdd9vqai=hGuQ8kuc9pgc9s8qqaq=dirpe0xb9q8qiLsFr0=vr0=vr0dc8meaabaqaciaacaGaaeqabaqabeGadaaakeaaiiGacuWFfpqDgaqeaaaa@2E92@_N _= number-weighted mean volume, υ¯
 MathType@MTEF@5@5@+=feaafiart1ev1aaatCvAUfKttLearuWrP9MDH5MBPbIqV92AaeXatLxBI9gBaebbnrfifHhDYfgasaacH8akY=wiFfYdH8Gipec8Eeeu0xXdbba9frFj0=OqFfea0dXdd9vqai=hGuQ8kuc9pgc9s8qqaq=dirpe0xb9q8qiLsFr0=vr0=vr0dc8meaabaqaciaacaGaaeqabaqabeGadaaakeaaiiGacuWFfpqDgaqeaaaa@2E92@_V _= volume-weighted mean volume, par = parenchyma, air = airspace, sep = septal tissue, alvepi = alveolar epithelium, alv = alveoli, typeII = type II cells, lb = lamellar bodies. Statistically significant differences between groups are indicated as: ^† ^p < 0.05 vs. group D0, ^|| ^p < 0.05 vs. group D3, ^§ ^p < 0.05 vs. group D6, ** p < 0.05 vs. group D9.

### Parameters related to quantification of pulmonary emphysema

A significantly higher alveolar number per lung was found after a duration of treatment of 3 weeks (Figure 3A) suggesting a lower degree of emphysematous alteration. Within the treated groups D3, D6 and D9 and the WT group, no statistically significant differences in alveolar number were observed. Consistent with a higher alveolar number, there was a significantly smaller mean alveolar volume in groups D3, D6 and D9 compared with D0 (Figure 3B). Again, the data did not show any differences between rfhSP-D treated groups, indicating similar dimensions in the distal airspaces. However, wild-type mice had significantly smaller alveoli. Thus, a longer period of treatment did not lead to a further attenuation of the degree of these indices of emphysema. These findings are consistent with a higher volume fraction of septal tissue within the lung parenchyma in groups D3, D6 and D9 compared to group D0. Unlike alveolar number and volume, the total surface area of alveolar epithelium per lung was not influenced by the treatment with rfhSP-D. The same was the case for the thickness of the alveolar septa. However, there was a significantly higher alveolar epithelial surface area per volume unit of parenchyma due to the treatment with rfhSP-D. Considering the volume fraction of alveolar space within parenchyma, no relevant differences could be observed, indicating that this parameter does not correlate with pulmonary emphysema.

In WT group, a rather high coefficient of variance could be observed regarding the alveolar number (CV_obs _= 0.36). As the coefficient of error of the alveolar number in this group is very small (CE = 0.07), the observed variance is mostly due to the biological variance of the given population and not due to a lack of precision of the stereological tool applied.

### Parameters related to type II cell alterations

Regarding the type II cell alterations, the data revealed a slightly but significantly smaller mean cell volume due to the treatment with rfhSP-D which did not depend on the period of treatment (Figure 4B). This reduction amounted to 12% on average but did not reach values within the range of type II cell sizes in the WT group. The number of type II cells per lung however, was slightly smaller in group D6 and was clearly reduced in group D9. Thus the effect on the number of type II cells demonstrated a dependence on the duration of treatment and was, compared to the WT group, normalized after 9 weeks of treatment (Figure 4A). In group D3 the number of type II cells per mm^3 ^parenchyma was slightly higher in comparison to group D0 but declined to lower values with longer periods of treatment.

### Parameters related to lamellar bodies

Compared to group D0 the volume fraction of lamellar bodies within type II cell was not significantly changed in group D3 but was significantly smaller in group D6 and D9. The same could be observed for the volume of lamellar bodies per cell, which was significantly lower in group D6 and D9 compared to group D0 but could not be normalized in respect to WT group (Figure 5A). The lamellar body content per lung was smaller, which was clearly related to the duration of treatment (Figure 5B). Group D6 contained significantly lower lamellar body volumes than group D0. Group D9, moreover, had a significantly lower volume of intracellular lamellar bodies than group D6 and was within the range of normal values. The volume-weighted mean volume of lamellar bodies was significantly smaller in group D6 compared to group D0 and D3 but did not significantly differ between the groups D0 and D3. The wild-type mice had the significantly lowest values compared to all other groups. The volume-weighted mean volume of lamellar bodies contains information on both mean particle size and variation in size [18,28]. Therefore, smaller values in this parameter after 6 weeks of treatment could be interpreted as a reduction of either the mean size of lamellar bodies or their variation in size or both.

## Discussion

Recent studies on SP-D have highlighted the protein's immunomodulatory function. In addition to its ability to enhance the clearance of pathogens, a dampening effect on inflammatory processes has become more apparent [7,8,31-34]. First applications of rfhSP-D to mice suffering from allergic asthma also showed a dampening of the allergic response due to allergen inhalation with a modification of cytokine levels and a decrease in airway hyper-responsiveness on allergen challenge [19,35]. Similar results were achieved in preterm newborn lambs which were exposed to LPS and treated with recombinant full length dodecamer SP-D or received no SP-D: in contrast to control animals the lambs which were treated with SP-D had stable lung function, a decreased systemic inflammatory response and survived [36]. Several patient groups show decreased levels of SP-D in their BAL, such as smokers [2,3], and patients with cystic fibrosis [4,5]. It is possible that the administration of recombinant SP-D might become a therapeutic option for patients suffering from diseases in which native SP-D levels are low [37,38]. Whilst the precise pathogenic impact of decreased SP-D levels in BAL in such diseases has not so far been clearly elucidated, it is clear that low SP-D levels are a feature of COPD, becoming more significant with years of smoking [3].

Native SP-D consists of four trimeric subunits. Within each subunit four domains can be distinguished: an N-terminal cysteine rich domain, a collagen-like domain, an α-helical coiled-coil neck domain and a CRD. Possible mechanisms for immunomodulation based on the CRD and collagen-like domain have been suggested recently [39]. Compared to native SP-D, the rfhSP-D used in the present study consists only of a small collagen-chain, the α-helical coiled-coil region and the CRD. Nevertheless, rfhSP-D expressed in E. coli possesses structural features that might be effective in binding ligands and recognizing immune cells, as revealed by high-resolution crystal structure analysis [20]. Furthermore, previous studies demonstrated its biological activity *in vivo*, although beneficial effects on lung structure have not been depicted so far [11,14,19,35].

The present study was performed to test the hypothesis whether the findings in SP-D knock-out mice after rfhSP-D treatment which demonstrate anti-inflammatory features of this truncated protein, were accompanied by an attenuation of the structural alterations present in SP-D deficient mice. The anti-inflammatory features which have been reported previously were characterized by decreased levels of chemokines such as MCP-1 and a diminished number of apoptotic and necrotic cells. The design of the present study included two variables: total dose and onset of treatment. Thus, the specific influence of each of these variables on the overall results can not be dissected, and the possibility that distinct developmental windows may differentially influence certain parameters can not be excluded. Our data provide evidence for the biological activity of rfhSP-D on the structural remodeling of the lung. In particular, we show that 1) the pulmonary emphysema is less severe, 2) both hyperplasia and hypertrophy of type II cells are less severe and 3) the excess intracellular surfactant pool is decreased due to rfhSP-D treatment.

With respect to the emphysema, we found a significantly higher alveolar number after only 3 weeks of treatment compared to the PBS treated D0 group. Compared to wild type mice, the number of alveoli was normalized. On the other hand the total surface area of alveolar epithelium was not influenced, independent of the duration of treatment. Former studies demonstrated that the alveolar surface area of human lungs compared to healthy lungs was only decreased in severe but not in moderate or mild emphysema so that this parameter is not sensitive enough to find slight emphysematous alterations [40]. Therefore, the total surface area of alveolar epithelium in the present study might not be appropriate as a parameter to distinguish between mild to moderate differences of the degree of emphysema. Moreover, in view of the fact that the treated mice have smaller lung volumes but the same alveolar surface area than the untreated mice it seems to be reasonable that the number of alveoli is higher in the treated mice: in smaller lungs the alveolar surface area is maintained by a higher number of alveolar septa. Consistent with this, we found a smaller mean alveolar volume, indicating smaller dimensions of the distal airspace as a result of the therapy with rfhSP-D.

As the higher number of alveoli after treatment with rfhSP-D is accompanied by a lower number of apoptotic and necrotic alveolar macrophages (which is also apparent after 3 weeks of treatment [14]) delayed corpse clearance with attendant consequences like increased ROS-production of bystander alveolar macrophages in SP-D knock-out mice might at least partly be responsible for the remodeling processes resulting in decreased alveolar numbers. Whether the increase in alveolar number after treatment results from a decreased destruction due to suppressed inflammatory activity or a generation of new alveoli or both is not clear. Wert et al. demonstrated the progressive character of the pulmonary emphysema in SP-D knock-out mice from the third week of live on [16]. As there was no difference in the degree of pulmonary emphysema between the three rfhSP-D treated groups, a generation of new alveoli as a result of the treatment with rfhSP-D can not be excluded. A previous study by Zhang et al. showed that a conditional expression of native rat SP-D was not able to correct an existing pulmonary emphysema according to qualitative findings, suggesting that the higher number of alveoli in our study is in part a consequence of an inhibition of alveolar destruction [41]. Kingma et al. provided evidence that the collagenous domain of SP-D is important to prevent mice lungs from emphysema development [42]. This study seems to contradict findings in the present study. Although rfhSP-D is missing a proper collagenous domain it was able to maintain lung structure, presumably by contributing to an anti-inflammatory environment. The anti-inflammatory effect of rfhSP-D can be explained by the model of Gardai et al. [39]. By binding of the CRD to the signal inhibitory regulatory protein α (SIRP α) rfhSP-D inhibits NFκB and consecutively immune cell activation and MMP-expression. Kingma et al. showed however that SP-D lacking the collagenous domain was not able to both generate an anti-inflammatory environment and prevent SP-D knock-out mice from developing pulmonary emphysema [42]. This contradiction to Gardai et al. [39] and other recently published data [11,14,19,33] was discussed as a consequence of an unanticipated change in the structure of the CRD [42]. Moreover rfhSP-D expressed in yeast which, opposed to E. coli expressed rfhSP-D, does not have any portion of the collagen region, was not able to prevent SP-D knock-out mice lungs from emphysema development after intranasal application (our unpublished observations), indicating a role of the expression-system for the biological activity of the fragment. Alternatively, the short collagen domain of rfhSP-D used in this study might be effective in maintaining its function. As previously shown, nearly 40% of the intranasally administered rfhSP-D could be detected one hour after injection in cell free lavage (4 μg rfhSP-D) [11]. The level of native SP-D in control mice amounts to 3 μg [11]. According to measurements in the study by Kingma et al. the levels of the collagenous domain deprived SP-D in BAL were 11 μg/ml [42]. Therefore a higher concentration of rfhSP-D in the present study is no appropriate explanation for the observed beneficial effect on lung structure.

Like the conditional replacement of native SP-D [41], the treatment with rfhSP-D led to lower surfactant phospholipid levels which, in case of the treatment with rfhSP-D, were significant after 6 weeks of therapy [14] demonstrating a reversibility of the disturbed surfactant homeostasis. In the present study, a decrease in the number of type II cells and the total volume of intracellular lamellar bodies per lung was not observed until after the same period of treatment, namely 6 weeks. These parameters were normalized after 9 weeks of treatment. This different behavior of the number of type II cells which declines after 6 weeks of treatment and the number of alveoli which is already influenced after 3 weeks of treatment might be a consequence of recently developed concepts, stating that the pulmonary emphysema and the alterations relating to type II cell and intracellular surfactant homeostasis are mediated by different mechanisms [9,43]. The treatment with rfhSP-D seems to influence both mechanisms. Botas et al. first observed alterations of type II cells in SP-D knock-out mice in the third week of life, indicating a progress of these alterations with age [10]. The fact that the type II cell number in knock-out mice is normalized even if treatment with rfhSP-D begins after the third week of life, suggests that the proliferation of these cells may be inhibited by the truncated SP-D-fragment. Alternatively, the anti-inflammatory effects of rfhSP-D may limit epithelial damage and subsequent epithelial repair.

The stereological findings in mice treated with rfhSP-D with respect to the type II cell alterations, as well as the intracellular surfactant pool, are similar to results seen in SP-D knock-out mice with additional ablation of the GM-CSF-gene [9]. The absence of both GM-CSF and SP-D in transgenic mice brought about a reduction of type II cell volume and number per lung, as well as a correction of the total volume of lamellar bodies per type II cell and lung, indicating that the alterations with respect to type II cells and the intracellular surfactant pool due to SP-D-deficiency are at least partly mediated by GM-CSF [9,43]. Consistently, previous studies in mice over-expressing GM-CSF showed a type II cell hypertrophy and also a hyperplasia [44]. Fisher et al. found that local recombinant rat SP-D expression in the lungs of SP-D knock-out mice was able to inhibit the synthesis of saturated phosphatidylcholine by type II cells, meaning that in SP-D-deficient mice the synthesis of surfactant material is increased [45]. The exact mechanism of this inhibition is not known but it is possible that it is a secondary effect due to a decrease of moderately elevated GM-CSF levels in SP-D knock-out mice [9,43]. At any rate, the earlier the therapy with rfhSP-D starts, and with it presumably the inhibition of the synthesis of saturated phosphatidylcholine, the lower are the amounts of intracellular surfactant content. Moreover, the absence of giant lamellar bodies and the decrease in volume of the intracellular surfactant per lung and cell as a consequence of rfhSP-D therapy could be considered a result of a reduced inflammatory state.

## Conclusion

The present study demonstrates that rfhSP-D treatment in SP-D knock-out mice results in beneficial effects on lung morphology. Even after a relatively late start of treatment in the ninth week of life, the degree of pulmonary emphysema could be reduced, as indicated by higher numbers of alveoli. A punctual start of the therapy in the third week of life was able to normalize the number of type II cells as well as the disturbances related to the intracellular pool of lamellar bodies. Thus, our data support the concept that patients suffering from lung diseases with decreased levels of SP-D in BAL such as COPD due to smoking or cystic fibrosis, might benefit from a therapy based on recombinant SP-D.

## Abbreviations

SP-D = surfactant protein D, SP-A = surfactant protein A, rfhSP-D = truncated fragment of human surfactant protein D, CRD = carbohydrate recognizing domain, BAL = bronchoalveolar lavage, V = volume, V_V _= volume fraction, S = surface area, S_V _= surface area density, τ¯
 MathType@MTEF@5@5@+=feaafiart1ev1aaatCvAUfKttLearuWrP9MDH5MBPbIqV92AaeXatLxBI9gBaebbnrfifHhDYfgasaacH8akY=wiFfYdH8Gipec8Eeeu0xXdbba9frFj0=OqFfea0dXdd9vqai=hGuQ8kuc9pgc9s8qqaq=dirpe0xb9q8qiLsFr0=vr0=vr0dc8meaabaqaciaacaGaaeqabaqabeGadaaakeaaiiGacuWFepaDgaqeaaaa@2E90@ = mean thickness, N = number, N_V _= numerical density, υ¯
 MathType@MTEF@5@5@+=feaafiart1ev1aaatCvAUfKttLearuWrP9MDH5MBPbIqV92AaeXatLxBI9gBaebbnrfifHhDYfgasaacH8akY=wiFfYdH8Gipec8Eeeu0xXdbba9frFj0=OqFfea0dXdd9vqai=hGuQ8kuc9pgc9s8qqaq=dirpe0xb9q8qiLsFr0=vr0=vr0dc8meaabaqaciaacaGaaeqabaqabeGadaaakeaaiiGacuWFfpqDgaqeaaaa@2E92@_N _= number-weighted mean volume, υ¯
 MathType@MTEF@5@5@+=feaafiart1ev1aaatCvAUfKttLearuWrP9MDH5MBPbIqV92AaeXatLxBI9gBaebbnrfifHhDYfgasaacH8akY=wiFfYdH8Gipec8Eeeu0xXdbba9frFj0=OqFfea0dXdd9vqai=hGuQ8kuc9pgc9s8qqaq=dirpe0xb9q8qiLsFr0=vr0=vr0dc8meaabaqaciaacaGaaeqabaqabeGadaaakeaaiiGacuWFfpqDgaqeaaaa@2E92@_V _= volume-weighted mean volume, par = parenchyma, air = airspace, sep = septal tissue, alvepi = alveolar epithelium, alv = alveoli, typeII = type II cells, lb = lamellar bodies, ROS = reactive oxygen species, GM-CSF = granulocyte macrophage-colony stimulating factor, MMP = matrix metalloproteinases, COPD = chronic obstructive pulmonary disease, MCP-1 = monocyte chemoattractant protein 1.

## Competing interests

The author(s) declare that they have no competing interests.

## Authors' contributions

All authors were involved in the conception, conduction and analysis of this study. All authors read and approved the final version of this manuscript.

LK carried out stereological analyses on light microscopic level

MO wrote about the stereological methods in "Material and Methods"

RM carried out both preparation and fixation of the mice lungs

PT and RD were responsible for the treatment of the mice with rfhSP-D or PBS

CM and FG carried out the stereological analyses on electron microscopic level

JR was responible for processing of the mice-lungs, including sampling and embedding

SH delivered the gene-targeted mice for analyses

KR delivered the rfhSP-D for mice treatment

HC wrote about the "Preparation of rfhSP-D" as well as major parts of "Background" and "Discussion"
